# Genetic variation of *Sporothrix globosa* isolates from diverse geographic and clinical origins in China

**DOI:** 10.1038/emi.2017.75

**Published:** 2017-10-11

**Authors:** Lipei Zhao, Yan Cui, Yu Zhen, Lei Yao, Ying Shi, Yang Song, Ruili Chen, Shanshan Li

**Affiliations:** 1 Department of Dermatology and Venereology, The First Hospital of Jilin University, Changchun, Jilin 130021, China

**Keywords:** amplified fragment length polymorphism, genotyping, sporotrichosis, *Sporothrix globosa*

## Abstract

*Sporothrix globosa* is the main causative agent of sporotrichosis, a common mycosis that usually affects the skin, in China. Despite increasing efforts in the molecular identification of this fungal pathogen, its modes of transmission and epidemiology remain poorly understood. The goals of this study were to assess the genetic diversity of *S. globosa* using amplified fragment length polymorphism (AFLP) analysis and to assess the correlation of AFLP profiles with the geographic origins, growth rates, clinical forms, and antifungal susceptibilities of *S. globosa* isolates. AFLP analysis of 225 clinical *S. globosa* isolates from eight provinces or municipalities in China identified eight distinct clustering groups (I–VIII), with groups I, II and IV being the most common. The AFLP genotypes showed distinct distribution patterns among different regions within Jilin Province and between northern and southern China, but there was no obvious association between the AFLP genotypes and the growth rates, clinical forms or antifungal susceptibilities of the *S. globosa* isolates. These results expand our understanding of the genetic variation of *S. globosa* and suggest that AFLP analysis is a potentially useful tool for studying the epidemiology of this fungal pathogen.

## INTRODUCTION

Sporotrichosis is a common chronic deep mycosis caused by the dimorphic fungus *Sporothrix schenckii.*^1^ Based on its clinical manifestations, sporotrichosis can be classified into fixed cutaneous, lymphocutaneous, disseminated cutaneous and extracutaneous forms. Sporotrichosis was first described in 1898 in the United States^2^ and has since been reported worldwide, with a high prevalence in tropical and subtropical areas. While *S. schenckii* has long been considered a single species, increasing numbers of phenotypic and molecular studies suggest that the pathogenic *Sporothrix* species comprises at least four closely related but clearly distinct species, including *S. schenckii sensu stricto*, *S. globosa, S. brasiliensis* and *S. luriei*.^3, 4^ Among them, *S. globosa* is perhaps the most extensively studied, with reports from North, Central, and South America, Europe and Asia.^5, 6^

Since the first report of sporotrichosis in China in 1916, the incidence of the disease has continued to increase. Several outbreaks have been reported, particularly in Jilin Province, where the largest number of sporotrichosis cases in China have been recorded.^7, 8^ Many molecular studies have demonstrated that *S. globosa* is the most prevalent etiologic agent of sporotrichosis in China.^8, 9, 10, 11, 12^ Several retrospective studies of clinical *S. schenckii* isolates from China using random amplified polymorphic DNA analysis^13, 14^ and restriction fragment length polymorphism analysis^15^ have shown a correlation among the genotypes, clinical forms, and geographic origins of isolates, although *S. schenckii* was not differentiated from *S. globosa* or other *Sporothrix* species.

Amplified fragment length polymorphism (AFLP) analysis is a highly sensitive method for detecting DNA polymorphisms and has been widely used for genetic variation and linkage analysis of bacteria, plants, and animals as well as fungi. When this technique was used to examine the genetic diversity of *S. schenckii* isolates in Peru, two distinct clusters were noted, although there was no correlation between these AFLP genotypes and the geographical origins or clinical manifestations of the disease.^16^ Recently, Zhang *et al.*^17^ applied AFLP analysis to 20 *S. globosa* isolates of diverse geographic origins, including nine isolates from China, and found that all isolates were tightly clustered into the same group. However, the study gave no detail on the genetic diversity of the nine isolates from China, and any geographic and phenotypic associations were not reported.

In the present study, we used AFLP analysis to examine 225 clinical *S. globosa* isolates from eight provinces or municipalities in China with the aim of identifying any correlations between AFLP profiles and the geographic origins, growth rates, clinical characteristics and antifungal susceptibilities of the isolates.

## MATERIALS AND METHODS

### Fungal isolates and cultivation

**Figure 1:**
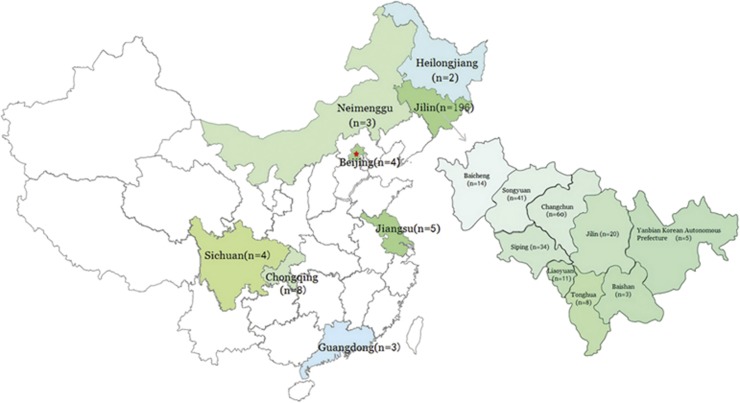
Geographical origins of the *Sporothrix globosa* isolates studied in this work.

A total of 225 clinical *S. globosa* isolates from China were included in this study. The isolates were collected between 2009 and 2013 from patients with sporotrichosis (one isolate per patient). The samples were collected at six hospitals, with the patients originating from eight different provinces or municipalities in China (Figure 1 and Table 1). Twenty four of the isolates had been previously identified as *S. globosa* based on sequence analysis of the calmodulin gene (*CAL*; Shiying, 2015, unpublished data). These sequences are available from the GenBank database using the accession numbers listed in Table 1. Demographic information and clinical manifestation data for each patient were provided by the investigators at each hospital (Table 1). *Aspergillus fumigatus* strain IFM40808 (wild-type) and three *S. globosa* isolates (ATCC MYA-4911, ATCC MYA-4912, and ATCC MYA-4914) were used as controls for AFLP analysis. These strains were provided as a gift by the Chinese Academy of Medical Sciences and Peking Union Medical College. All isolates were inoculated onto potato dextrose agar slants and cultured at 28 °C for 7 days.

### Morphological and physiological studies

Samples of mycelia (~1 mm diameter) from each culture slant were subcultured on fresh potato dextrose agar plates and incubated at various temperatures (30 °C, 35 °C or 37 °C) for 21 days. Colony diameters were measured after 7, 14 and 21 days of incubation. Assimilation of carbon sources, including sucrose and raffinose, was examined according to previously described methods.^18^ All isolates were assayed in 96-well microplates, and each plate contained positive controls with glucose and negative controls with no carbon source. Conidial viability in the presence of the different carbon sources was determined following incubation for 5 days at 25 °C.

### DNA extraction and sequencing

Total genomic DNA of all isolates was extracted using an alkaline lysis extraction method.^19, 20^ Briefly, fungal pellets were resuspended sequentially in Solution I (0.9% w/v glucose, 25 mmol/L Tris, 6 mmol/L ethylenediaminetetraacetic acid, pH 8.0) and Solution II (1% SDS, 0.2 mol/L sodium hydroxide), followed by precipitation with Solution III (12% sodium acetate, 12% acetic acid). The supernatant was collected and successively treated with phenol:chloroform:isoamyl alcohol (25:24:1) and chloroform:isoamyl alcohol (24:1). Nucleic acids were precipitated with ice-cold isoamyl alcohol at −20 °C. DNA pellets were washed with 70% ethanol, dissolved in 40 μL of ddH_2_O, and stored at −20 °C until use.

All DNA samples were quantified using an ultraviolet spectrophotometer, and then the quality was checked using 0.8% (w/v) agarose gel electrophoresis. To confirm that an isolate was *S. globosa*, we performed a PCR assay to amplify the *S. globosa CAL* gene using the previously reported primers CL1 (5′-GA(GA)T(AT)CAAGGAGGCCTTCTC-3′) and CL2A (5′-TTT TTG CAT CAT GAG TTG GAC-3′).^21^ The thermocycling conditions included an initial denaturation at 94 °C for 1 min, followed by 35 cycles of 94 °C for 30 s, 60 °C for 30 s, and 72 °C for 1 min, and a final extension of 72 °C for 5 min. The PCR products were examined by electrophoresis in 0.8% (w/v) agarose gels. In addition, all products were purified and subjected to Sanger sequencing by Sangon Biotech (Shanghai, China).

### AFLP analysis

The AFLP procedure was carried out essentially as described by Vos *et al.*^22^ with some modifications. All primers and adapters^22^ were synthesized by Sangon Biotech. Briefly, 1 μg of genomic DNA was digested with FastDigest *Eco*RI (1 μL) and FastDigest *Saq*AI (1 μL; an isoschizomer of *Mse*I) in a 20- μL reaction mixture at 37 °C for 5 min. The digested products were then ligated to their respective adapters (*Eco*RI adapter, 5′-CTCGTAGACTGCGTACC-3′ *Saq*AI adapter, 5′-GACGATGAGTCCTGAG-3′) using T4 DNA Ligase (Invitrogen, Carlsbad, CA, USA) at 25 °C for 1 h. The quality and quantity of the digested and ligated products were examined by agarose gel electrophoresis.

Preamplification was performed in a total volume of 20 μL, containing 5 μL of diluted (1:20) ligation products, 2 mM magnesium chloride, 0.2 mM of each dNTP, 2 μL of 10 × PCR buffer, 1 U *Taq* DNA polymerase (Takara, Otsu, Japan), and 1 μL of each primer (*Eco*RI-A, 5′-GTA GAC TGC GTA CCA ATT CA-3′ *Saq*AI-C, 5′-GAC GAT GAG TCC TGA GTA AC-3′ 10 μM). The thermocycling conditions were: 94 °C for 2 min, followed by 25 cycles of 94 °C for 30 s, 56 °C for 1 min, and 72 °C for 1 min, and a final extension of 72 °C for 10 min.

Selective amplification was carried out in a 20- μL reaction volume consisting of 5 μL of diluted (1:20) preamplification products, 1 mM magnesium chloride, 0.15 mM of each dNTP, 2 μL of 10 × PCR buffer, 1.5 U *Taq* DNA polymerase (Takara), and 0.6 μL of each primer (*Eco*RI-ACT, 5′-GAC TGC GTA CCA ATT CAC T-3′ *Saq*AI-CAA, 5′-GAT GAG TCC TGA GTA ACA A-3′ 10 μM). The thermocycling conditions were as follows: 94 °C for 4 min, followed by 12 cycles of 94 °C for 30 s, 65 °C (with a 0.7 °C decrease per cycle) for 1 min, and 72 °C for 1 min, and then 23 cycles of 94 °C for 30 s, 56 °C for 1 min, and 72 °C for 1 min. The products of the selective amplification were separated by 6% (w/v) denaturing polyacrylamide gel in 1 × TBE buffer for ~1.5 h at 50 W. Following staining with 2% (w/v) silver nitrate, the gels were scanned with a Bio-Rad gel imaging system (Hercules, CA, USA), and the DNA bands were manually scored as present (1) or absent (0) and compiled into a binary matrix. The raw data were analyzed using the unweighted pair-group method with arithmetic average and the Dice coefficient, as implemented in NTSYS-pc version 2.10 (Exeter Software Co., Setauket, NY, USA).

### Antifungal agents and antifungal susceptibility testing

We assessed the susceptibility of 43 of the *S. globosa* isolates (Table 1), which represented each of the different AFLP genotypes, to eight antifungal agents, including amphotericin B (AMB; Bio Basic Inc., Markham, ON, Canada), terbinafine (TRB), itraconazole (ICZ), fluconazole (FCZ), voriconazole (VCZ), posaconazole (POS), albaconazole (ALB) and caspofungin (CAS). TRB, ICZ, FCZ and VCZ were purchased from Tokyo Chemical Industry, Tokyo, Japan. POS, ALB, and CAS were purchased from Toronto Research Chemicals, Toronto, ON, Canada. All susceptibility assays were carried out in RPMI 1640 medium buffered to pH 7 with 0.165 mol/L morpholinepropanesulfonic acid (MOPS). The *S. globosa* isolates were cultured in microplates, which were prepared as described by the Clinical and Laboratory Standards Institute (standard M38-A2).^23^ Final drug concentrations ranged from 0.125–64 μg/mL for FCZ, and from 0.03–16 μg/mL for the other drugs. Each inoculum was prepared by adding 5 mL of sterile saline to the agar plate and then removing the colony surface by gentle scraping. The resulting suspensions were diluted, and the numbers of conidia in the suspensions were adjusted to twice the desired final concentration ((1–5) × 10^4^ colony-forming units/mL). The microplates were incubated at 30 °C and read after 72 h. Minimum inhibitory concentrations (MIC) were determined as per the guidelines of the Clinical and Laboratory Standards Institute (standard M38-A2).^23^ *Candida parapsilosis* ATCC 22019 and *Candida krusei* ATCC 6258 were used as quality control strains in the antifungal susceptibility testing assays.

### Statistical analysis

Analysis of variance (ANOVA) and Dunnett’s T3 Test were used to evaluate the differences in the growth rates, colony sizes, and MIC values of isolates grown under different conditions, and the relationship between clinical manifestation and colony size. The chi-square test and Fisher’s exact test were used to evaluate the relationships between AFLP profiles and the geographic origin, sex and age of patients, clinical manifestation, and year of identification. All statistical analyses were performed using SPSS software version 21 (IBM SPSS Statistics, Somers, NY, USA). A value of *P*<0.05 was considered statistically significant. The reliability of AFLP clustering analysis was evaluated using a high cophenetic correlation coefficient after 1000 permutations (*r*=0.772).

## RESULTS

### Morphological and physiological analyses

All isolates demonstrated good growth by 21 days of cultivation on potato dextrose agar at 30 °C and 35 °C. All isolates initially produced cream-colored colonies, some of which gradually deepened in color to brown or black. Most colonies were oval or round in shape, with a wrinkled surface and a milky membranous edge. The colony diameters were 16–42 mm at 30 °C and 3–15 mm at 35 °C. When the culture temperature was increased to 37 °C, most isolates showed very limited growth, with colony sizes ranging from 1.5–5.5 mm in diameter. Seven isolates showed no growth at this temperature (FHJU12030101, FHJU12010502, FHJU12010402, FHJU11050201, FHJU12062301, FHJU11102601 and CCMC1). All growth data are summarized in Table 2. ANOVA and Dunnett’s T3 Test results showed that the average colony size of AFLP group IV isolates was significantly different from those of group I and group II isolates, while no significant difference in the average colony size was observed between any other AFLP groups. There were no significant differences (*P*>0.05) in the average colony size between isolates grown at different temperatures or between isolates obtained from patients with different clinical forms of sporotrichosis. All isolates assimilated glucose and sucrose, but none could assimilate raffinose.

### Sequencing of the *S. globosa CAL* gene

High-quality DNA (*OD*_260_/*OD*_280_ ratio values of 1.8–2.0) was extracted from all isolates, and the *CAL* gene was successfully amplified in all cases. The resulting ~770 bp amplicons were sequenced and subjected to BLAST analysis against the GenBank database. All sequences showed 99%–100% nucleotide sequence identity to *CAL* from *S. globosa* type strain CBS 120340, confirming that all isolates were *S. globosa*. The sequences generated in this study have been deposited in GenBank under the accession numbers shown in Table 1.

### AFLP profile and correlation analysis

**Figure 2:**
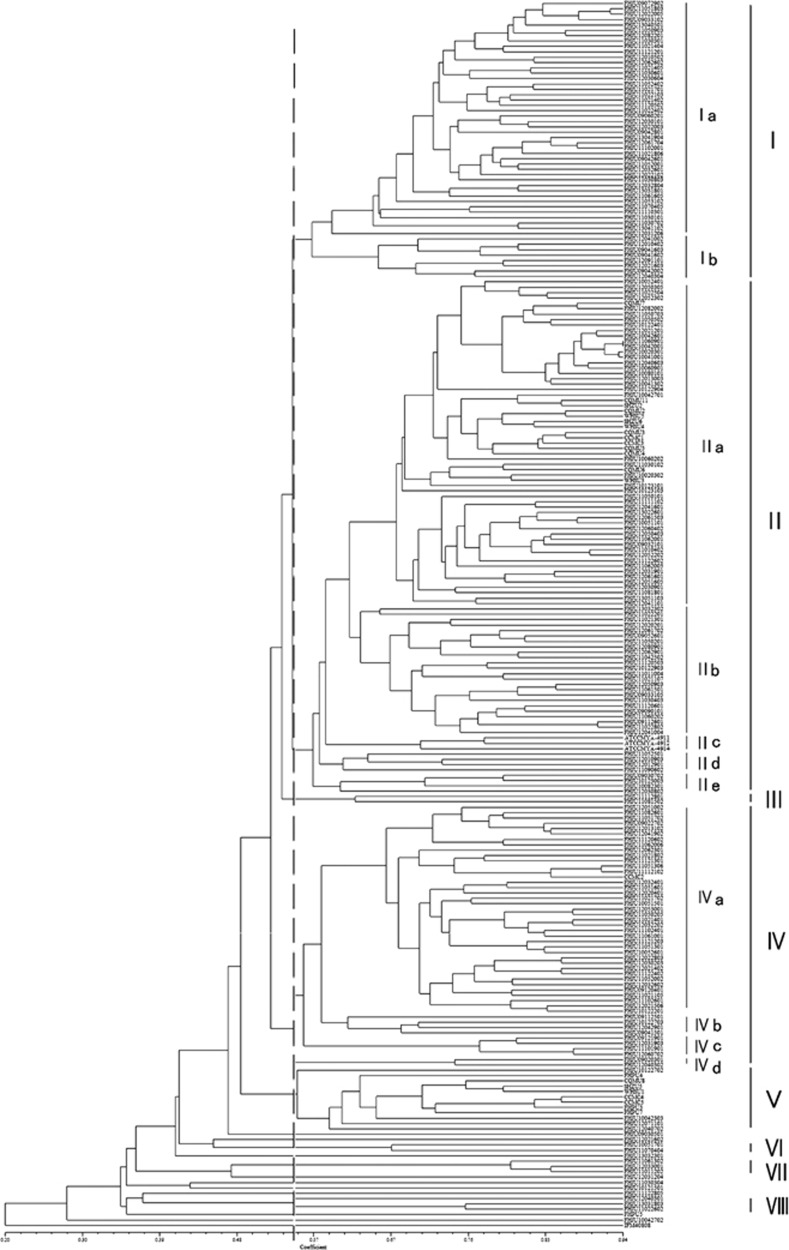
Clustering dendrogram of the 225 *Sporothrix globosa* isolates based on amplified fragment length polymorphism profiles generated using the unweighted pair-group method with arithmetic mean and the Dice coefficient. Eight major groups (designated I–VIII) were obtained at a coefficient of 0.55.

The AFLP profiles of the 225 *S. globosa* isolates and four reference strains are shown in Figure 2. A total of eight main clustering groups (designated I–VIII) were identified at a cophenetic correlation coefficient of 0.55. Nine isolates (FHJU11122805, FHJU12021602, FHJU13032301, FHJU12031204, FHJU11030304, FHJU10121501, FHJU10042702, FHJU09030501 and FHPU5) failed to form clusters and were well separated from the eight main clustering groups. Groups I, II, and IV could each be divided into a further two or five subgroups (Figure 2). The AFLP profile similarity levels among these 229 isolates ranged from 0.20 to 0.94. Group II was the most prevalent group, accounting for 42% of all isolates, followed by group I (23%) and group IV (21%). Three *Sporothrix globosa* reference isolates were clustered into Group II, and the *A. fumigatus* control isolate deviated from all *S. globosa* isolates. All the remaining groups were much less prevalent (no more than 5%).

The majority of the isolates involved in this study originated from nine different regions within Jilin Province (*n*=196; 86%). As shown in Table 3, isolates from Changchun (*n*=60) belonged to AFLP groups II (35/60) and IV (25/60). The Siping isolates mainly belonged to group II (23/31), while the Baicheng isolates (*n*=12) and most of the isolates from Jilin City (18/19) were clustered together into group I. Groups III and VIII consisted entirely of isolates from Songyuan. A significant association was found between AFLP profiles and geographic origins within Jilin Province (*χ*^2^-test, *P*=0.000, Table 3).

The remaining 28 isolates originated from seven other provinces or municipalities, and most (25/28, 89%) clustered into groups IIa and V. When comparing isolates from northern China (including Jilin, Heilongjiang, Neimenggu and Beijing) and southern China (including Jiangsu, Sichuan, Chongqing and Guangdong), the isolates from northern China primarily clustered in groups I, II and IV (52/196, 26.5% 79/196, 40.3% and 48/196, 24.5%, respectively), while the isolates from southern China mostly clustered in groups IIa, IVa and V (14/20, 70% 1/20, 5% and 5/20, 25%, respectively). Statistical analysis showed a significant difference in the distribution of the AFLP genotypes between northern and southern China (*χ*^2^-test, *P*=0.000, Table 3).

We attempted to correlate the AFLP profiles with the clinical forms of sporotrichosis (Table 4) but observed no significant correlation (*P*=0.251). In addition, Fisher’s exact test showed no significant association between the AFLP profiles and the sex (*P*=0.159) or age (*P*=0.565) of the patients or the sampling dates (*P*=0.052).

### Antifungal susceptibility testing

Antifungal susceptibility testing results are presented in Table 5. Of the eight drugs tested, TRB showed the strongest anti- *S. globosa* activity, with MIC values ranging from 0.03 to 8 μg/mL (geometric mean, 0.05 μg/mL), followed by POS, which produced MIC values ranging from 0.5 to >16 μg/mL (geometric mean, 2.99 μg/mL). Moderate anti- *S. globosa* activity was observed for CAS, ALB and ICZ, with MIC values ranging from 0.25 to >16 μg/mL, 4 to 16 μg/mL, and 1 to >16 μg/mL, respectively. FCZ, VCZ and AMB showed poor activity against *S. globosa*.

Statistical analysis showed that the observed MIC values were not associated with the AFLP genotypes, the origins of the isolates, or the clinical manifestations of the infection (ANOVA and Dunnett’s T3 Test, *P*>0.05, Table 5).

## DISCUSSION

In the present study, we examined the growth characteristics of 225 *S. globosa* isolates from sporotrichosis patients originating from eight provinces or municipalities in China. Using AFLP analysis, we categorized the isolates into eight distinct clustering groups. We also examined whether there was any correlation between the AFLP profiles and the *in vitro* growth characteristics, antifungal susceptibility, geographic origins and clinical forms of sporotrichosis.

AFLP analysis of the 225 *S. globosa* isolates in the current study showed that the AFLP genotypes had certain associations with the geographical origins of the isolates, especially those from Jilin Province. In particular, the isolates from Baicheng and Jilin City mostly clustered into group I, while isolates from Siping mainly clustered in group II. Isolates from Changchun were clustered into two groups: those from the central area clustered into group II, and isolates from areas near the border were identified as group IV. However, isolates from other regions, such as Songyuan and Liaoyuan, showed a great variety of genotypes. The reason for this genetic variation is unclear, but we suspect that it may be related to the frequent migration of the people in these regions. In addition, in contrast to the isolates from northern China, which were primarily clustered in groups I, II and IV, the isolates from southern China mainly clustered in groups IIa, V and IVa. While further studies are needed using a larger number of samples (especially from southern China), this observation agrees with Zhang *et al.*,^15^ who found a significant difference in the restriction fragment length polymorphism and Southern blotting band patterns of *S. globosa* isolates from southern and northern China. In contrast, Zhang *et al.*^17^ recently reported an AFLP analysis of 20 *S. globosa* isolates from wide geographic origins, wherein all of the isolates showed an identical AFLP pattern. The reasons for these discrepancies may include different experimental conditions or differences in the sample sizes. Different restriction endonucleases and different numbers of selective bases and primer pairs will influence the observed genetic diversity. For example, Neyra *et al.*^16^ used the restriction endonucleases *Eco*RI and *Mse*I, along with a combination of six primers, in their AFLP analysis of Peruvian strains of *S. schenckii*, identifying two stable populations. Zhang *et al.*^17^ used the same restriction enzymes but only one selective primer pair to divide 122 strains of *Sporothrix* into 13 groups. In the present study, we chose the same restriction endonucleases and one pair of primers (out of 64 primer pairs screened) and achieved reproducible results with a relatively high number of polymorphic bands.

In the current study, the AFLP genotypes appeared to be differentially distributed among different years. For example, AFLP group I accounted for the largest proportion of isolates in 2009 (8/20, 40%) and 2013 (4/8, 50%) but was absent in 2010, while group II was dominant in 2010 (18/25, 72%) but decreased in prevalence in 2011 (27/77, 35.1%) and 2012 (25/63, 39.7%). However, these differences did not reach statistical significance. Further studies are needed to confirm this observation using a larger number of isolates over a longer period of time.

All of the *S. globosa* isolates in the current study grew well at both 30 °C and 35 °C, with 97% (218/225) of isolates showing some growth at 37 °C, although the average growth rate and colony size were decreased compared with those at the lower temperatures (Table 2). Only seven isolates could not grow at 37 °C. These observations are consistent with the report of Yu *et al.*^10^ but contradict the report of Marimon *et al.*,^18^ who found that *S. globosa* did not grow at 37 °C, with the exception of four strains that produced colonies of 2 mm in diameter. There have been conflicting reports regarding the relationship between temperature sensitivity and clinical forms of *Sporothrix* infection. Kwon-Chung^24^ reported that isolates causing lymphocutaneous and extracutaneous sporotrichosis grew well at both 35 °C and 37 °C, whereas isolates causing fixed cutaneous sporotrichosis grew well at 35 °C but failed to grow at 37 °C. However, Yu *et al.*^10^ found that isolates obtained from the three cutaneous forms of sporotrichosis were able to grow at 37 °C. In our study, all but seven of the 225 isolates showed growth at 37 °C. The seven isolates that did not grow included four isolates associated with lymphocutaneous sporotrichosis, two from fixed cutaneous sporotrichosis, and one (CCMC1) from an undefined form of sporotrichosis. We found no significant association between the clinical form of the disease and the AFLP genotype or growth rate of *S. globosa*. Hence, the clinical manifestation of sporotrichosis is most likely related to the immune status of the patient rather than the thermotolerance or AFLP genotype of the causative *S. globosa* strain.

In the present study, we examined the drug susceptibilities of 43 *S. globosa* isolates representing each of the AFLP genotypes and did not detect any significant association between the *in vitro* antifungal drug susceptibility and the AFLP genotype. Nevertheless, our data indicated that all *S. globosa* isolates are highly sensitive to TRB, consistent with previous studies.^25, 26^ Although only two of the examined isolates belonged to group VI, both were more sensitive to TRB than to any of the other drugs, including ICZ. Ten isolates (23%) showed resistance to POS, with MIC values of ⩾16 μg/mL. Moreover, AMB showed poor activity against all isolates, which is in contrast to previous studies.^12, 25^ These findings suggest a need to determine the antifungal susceptibility of *S. globosa* isolates in China on a larger scale to optimize the treatment of sporotrichosis.

In summary, the current AFLP analysis revealed significant genetic diversity among *S. globosa* isolates in China. The AFLP profiles of the isolates are associated with their geographic origins, but not with other phenotypic properties of the isolates. This study suggests that AFLP analysis is a potentially useful tool for studying the epidemiology of *S. globosa.* Further studies using a larger number of *S. globosa* isolates from patients from wider geographic origins and suffering from more diverse well-defined clinical forms of sporotrichosis are required to better understand the implications of the high degree of AFLP variation in the epidemiology of sporotrichosis.

## Figures and Tables

**Table 1:** Clinical isolates of *Sporothrix globosa* included in the study

	**Location**	**Isolate ID**^a^	**Year of identification**	**Gender (M/F)**	**Age (year)**	**Clinical form (D, F, L, E)**^b^	**GenBank Accession code**
Jilin Province	Baicheng City	FHJU09033102^c^	2009	M	4	F	KY349948
		FHJU09060201	2009	F	56	L	KY349946
		FHJU09072902	2009	F	42	F	KY349944
		FHJU10042702	2010	M	41	L	KY350105
		FHJU11021404	2011	F	11	L	KY349942
		FHJU11030301	2011	F	49	L	KY349947
		FHJU11051803	2011	M	0.25	F	KY350125
		FHJU11053102^c^	2011	F	57	D	KY350126
		FHJU11070405	2011	F	3	F	KY349943
		FHJU11110301	2011	F	43	F	KY349940
		FHJU11122805	2011	M	34	F	KY349945
		FHJU12022005	2012	F	15	L	KY349934
		FHJU12061704	2012	F	44	F	KY349939
		FHJU13041904	2013	F	53	F	KY349941
	Baishan City	FHJU09042601	2009	F	54	F	KY349965
		FHJU10122702^c^	2010	F	24	F	KY350091
		FHJU13031801^c^	2013	F	51	F	KY349978
	Changchun City	FHJU09030702	2009	M	9	F	KY350078
		FHJU09032101	2009	M	57	F	KY350061
		FHJU09041501	2009	M	13	F	KY350075
		FHJU09112501	2009	F	56	L	KY350073
		FHJU09120401	2009	F	53	L	KY350068
		FHJU09121901	2009	F	29	F	KY350046
		FHJU10041001	2010	F	9	F	KY350123
		FHJU10041302	2010	F	73	L	KY350085
		FHJU10042601	2010	F	38	L	KY350116
		FHJU10042701	2010	F	27	L	KY350110
		FHJU10052601	2010	F	17	L	KY350109
		FHJU10122201	2010	M	5	F	KY350088
		FHJU10122703	2010	F	2	F	KY350090
		FHJU10122903^c^	2010	F	74	L	KY350101
		FHJU10122904	2010	F	59	F	KY350086
		FHJU10123003	2010	F	4	F	KY350120
		FHJU10123103	2010	M	5	F	KY350092
		FHJU10082301^c^	2011	F	73	E	KY350117
		FHJU11010402	2011	F	60	F	KY350054
		FHJU11021105	2011	F	65	F	KY350060
		FHJU11021802	2011	M	58	F	KY350064
		FHJU11022504	2011	F	48	F	KY350058
		FHJU11030102	2011	F	53	L	KY350071
		FHJU11050205	2011	F	62	L	KY350133
		FHJU11050502^c^	2011	M	0.25	F	KY350094
		FHJU11050703	2011	F	4	L	KY350080
		FHJU11051301	2011	F	22	F	KY350098
		FHJU11051306	2011	F	47	L	KY350134
		FHJU11051601^c^	2011	F	77	L	KY350074
		FHJU11060901	2011	M	9	L	KY350097
		FHJU11061001	2011	F	28	F	KY350041
		FHJU11062001^c^	2011	F	56	D	KY350056
		FHJU11101901^c^	2011	M	78	F	KY350044
		FHJU11102601^c^	2011	F	59	F	KY350076
		FHJU11112102	2011	F	40	F	KY350072
		FHJU11121203	2011	F	39	L	KY350055
		FHJU12010903	2012	F	65	F	KY350059
		FHJU12012901	2012	F	45	F	KY350057
		FHJU12013003	2012	F	68	L	KY350077
		FHJU12020401	2012	F	42	F	KY350082
		FHJU12021201	2012	F	51	L	KY350084
		FHJU12021506	2012	F	54	L	KY350093
		FHJU12030802	2012	F	9	F	KY350040
		FHJU12031901	2012	F	46	F	KY350069
		FHJU12031903	2012	M	33	F	KY350042
		FHJU12032401	2012	F	60	L	KY350062
		FHJU12040603	2012	M	43	F	KY350095
		FHJU12041601	2012	F	62	L	KY350096
		FHJU12042901	2012	F	3	F	KY350081
		FHJU12050305	2012	F	48	F	KY350065
		FHJU12050403	2012	F	9	L	KY350039
		FHJU12052202	2012	F	79	F	KY350063
		FHJU12052302	2012	F	62	L	KY350079
		FHJU12060402	2012	F	45	L	KY350066
		FHJU12060702	2012	F	50	F	KY350043
		FHJU12061503	2012	F	47	F	KY350067
		FHJU12061601	2012	F	37	F	KY350083
		FHJU12062301	2012	F	6	F	KY350045
		FHJU12082002	2012	F	64	F	KY350070
		FHJU13032302	2013	M	17	F	KY350001
	Jilin City	FHJU09041602	2009	F	45	L	KY349981
		FHJU09041603^c^	2009	M	0.3	F	KY349980
		FHJU09042002	2009	M	44	L	KY349989
		FHJU09042801	2009	F	52	L	KY349967
		FHJU10051701^c^	2010	F	67	F	KY350112
		FHJU11030601	2011	M	9	F	KY349991
		FHJU11030803	2011	F	75	L	KY350129
		FHJU11052001	2011	M	4	F	KY349993
		FHJU11102001^c^	2011	M	4	F	KY349972
		FHJU12010402	2012	F	68	L	KY349982
		FHJU12010502	2012	F	59	L	KY349986
		FHJU12021602	2012	F	44	L	KY349987
		FHJU12021603	2012	F	12	F	KY349974
		FHJU12022003	2012	F	76	L	KY349985
		FHJU12030604	2012	F	5	F	KY349988
		FHJU12031206	2012	M	60	F	KY349983
		FHJU12032601	2012	M	6	F	KY349992
		FHJU12040304	2012	M	12	F	KY349990
		FHJU12062602	2012	F	54	L	KY349984
		FHJU12091101	2012	F	59	L	KY349973
	Liaoyuan City	FHJU09020301	2009	F	59	L	KY350008
		FHJU09052601	2009	M	61	L	KY350007
		FHJU10020301^c^	2010	F	55	L	KY350113
		FHJU10060901	2010	M	51	F	KY350099
		FHJU11021301	2011	F	46	F	KY349979
		FHJU11021806^c^	2011	M	48	D	KY349976
		FHJU11061302^c^	2011	F	70	L	KY350006
		FHJU11070404^c^	2011	M	59	L	KY350005
		FHJU11120503	2011	M	1.5	F	KY349994
		FHJU12061702	2012	F	28	L	KY350004
		FHJU13041102	2013	F	42	F	KY349975
	Siping City	FHJU09022702	2009	F	44	F	KY350127
		FHJU09030501	2009	F	7	F	KY350131
		FHJU09033105	2009	M	1	L	KY350011
		FHJU09090101	2009	F	59	F	KY350015
		FHJU09112601	2009	F	4	F	KY350020
		FHJU10042303^c^	2010	M	29	F	KY350121
		FHJU10051101	2010	M	2	F	KY350103
		FHJU10052401	2010	F	50	F	KY350107
		FHJU10080101	2010	F	36	F	KY350100
		FHJU10121501	2010	M	1	F	KY350087
		FHJU10123101	2010	F	41	F	KY350102
		FHJU11011004	2011	M	3	F	KY349996
		FHJU11011202^c^	2011	F	47	L	KY350034
		FHJU11021107	2011	F	6	L	KY350012
		FHJU11022201	2011	M	48	L	KY349998
		FHJU11022802	2011	F	67	L	KY350018
		FHJU11030403	2011	F	60	F	KY350014
		FHJU11042502	2011	F	19	L	KY350016
		FHJU11050201	2011	F	30	L	KY349999
		FHJU11060202	2011	M	28	F	KY350013
		FHJU11061501	2011	F	63	L	KY350017
		FHJU11082601	2011	F	60	F	KY350024
		FHJU11120601	2011	M	7	L	KY350021
		FHJU11120602	2011	M	7	L	KY350038
		FHJU12020201	2012	F	55	F	KY350023
		FHJU12022803	2012	M	2	F	KY350037
		FHJU12033001^c^	2012	M	82	L	KY350009
		FHJU12040302^c^	2012	F	70	F	KY349995
		FHJU12041004	2012	F	44	F	KY350130
		FHJU12050903	2012	M	55	L	KY350022
		FHJU12051605	2012	F	24	F	KY350010
		FHJU12062901	2012	F	4	F	KY350000
		FHJU12080901	2012	F	54	F	KY349997
		FHJU13032301	2013	M	14	L	KY350019
	Songyuan City	FHJU10020401	2010	F	47	L	KY350108
		FHJU10042001^c^	2010	M	3	F	KY350111
		FHJU10051501	2010	M	3	F	KY350104
		FHJU10060202^c^	2010	M	0.5	F	KY350106
		FHJU10122401	2010	M	5	F	KY350089
		FHJU11021402^c^	2011	F	52	D	KY349968
		FHJU11021405^c^	2011	F	48	D	KY349950
		FHJU11021701	2011	M	39	L	KY349954
		FHJU11022103	2011	M	4	F	KY349953
		FHJU11022402	2011	M	6	L	KY349952
		FHJU11022602^c^	2011	F	6	F	KY349955
		FHJU11030101	2011	M	7	L	KY349936
		FHJU11030304	2011	F	24	F	KY349949
		FHJU11030702	2011	F	54	F	KY349956
		FHJU11050903	2011	M	11	F	KY349938
		FHJU11051702	2011	F	62	L	KY350122
		FHJU11052002	2011	F	27	L	KY350032
		FHJU11052402	2011	F	62	L	KY350124
		FHJU11052501	2011	M	43	L	KY349958
		FHJU11061605	2011	M	48	L	KY349963
		FHJU11062006	2011	F	50	F	KY350028
		FHJU11081502^c^	2011	M	25	F	KY349970
		FHJU11090602	2011	F	58	F	KY349957
		FHJU11112801^c^	2011	M	55	F	KY349969
		FHJU11120502	2011	F	75	F	KY350132
		FHJU11121201	2011	F	29	L	KY349959
		FHJU12013102	2012	M	64	F	KY350036
		FHJU12021402	2012	F	62	L	KY350029
		FHJU12022102	2012	F	54	L	KY349971
		FHJU12030101^c^	2012	F	58	L	KY349964
		FHJU12030203^c^	2012	M	50	D	KY350030
		FHJU12030901	2012	M	43	F	KY350035
		FHJU12032602	2012	F	58	F	KY350031
		FHJU12032804	2012	F	5	F	KY349935
		FHJU12040301^c^	2012	F	53	L	KY349960
		FHJU12041002^c^	2012	F	45	F	KY349951
		FHJU12041902	2012	M	55	F	KY350033
		FHJU12051002	2012	F	64	F	KY349966
		FHJU12082201	2012	F	55	L	KY349937
		FHJU13031803^c^	2013	F	46	F	KY349962
		FHJU13040501	2013	M	10	F	KY349961
	Tonghua City	FHJU11021702	2011	M	36	F	KY350026
		FHJU11062005	2011	F	51	F	KY350128
		FHJU11081801^c^	2011	F	9	F	KY350053
		FHJU11111102	2011	M	44	L	KY350050
		FHJU11122402	2011	F	50	F	KY350027
		FHJU12053001	2012	F	49	F	KY350025
		FHJU13022601	2013	F	58	L	KY350003
		FHJU13051103^c^	2013	F	7	F	KY350002
	Yanbian Korean Autonomous Prefecture	FHJU11102401^c^	2011	F	3	F	KY350047
		FHJU11121301^c^	2011	F	77	L	KY350049
		FHJU11122602	2011	M	7	L	KY350118
		FHJU12031204	2012	F	68	L	KY350119
		FHJU12040702^c^	2012	M	65	F	KY350115
Neimenggu Autonomous Region	Tongliao City	FHJU11021401^c^	2011	F	41	F	KY350052
		FHJU12032202^c^	2012	F	60	L	KY350051
		FHJU12041101	2012	M	4	F	KY350048
Heilongjiang Province	Wuchang City	FHJU11050101^c^	2011	M	7	L	KY349977
	Hegang City	FHJU12071101^c^	2012	M	64	F	KY350114
Jiangsu		CMCC1					KR075722^d^
Jiangsu		CMCC2					KR075723^d^
Jiangsu		CMCC3					KR075724^d^
Jiangsu		CMCC4					KR075725^d^
Jiangsu		CMCC5					KR075726^d^
Chongqing		CQMU11					KR075728^d^
Chongqing		CQMU2					KR075729^d^
Chongqing		CQMU3					KR075730^d^
Chongqing		CQMU4					KR075731^d^
Chongqing		CQMU5					KR075762^d^
Chongqing		CQMU6					KR075732^d^
Chongqing		CQMU7					KR075763^d^
Chongqing		CQMU8					KR075733^d^
Beijing		FHPU3					KR075744^d^
Beijing		FHPU4					KR075745^d^
Beijing		FHPU5					KR075746^d^
Beijing		FHPU7					KR075748^d^
Guangdong		SHZU2					KR075750^d^
Guangdong		SHZU5					KR075753^d^
Guangdong		SHZU6					KR075754^d^
Sichuan		WHSU1					KR075758^d^
Sichuan		WHSU3					KR075759^d^
Sichuan		WHSU4					KR075760^d^
Sichuan		WHSU5					KR075761^d^

Abbreviations: female, F; male, M.

a The first four alphabet letters in the isolate ID represent abbreviations for the following hospitals: FHJU, The First Hospital of Jilin University; CCMC, Institute of Dermatology, Chinese Academy of Medical Sciences and Peking Union Medical College; CQMU, the First Affiliated Hospital of Chongqing Medical University; FHPU, Peking University First Hospital; SHZU, Second Affiliated Hospital of Zhongshan University; WHSU, West China Hospital of Sichuan University.

b F—fixed cutaneous; L—lymphocutaneous; D—disseminated cutaneous; E—extracutaneous.

c Isolates which performed the antifungal susceptibility.

d These 24 isolates were previously identified as *S. globosa* by Shiying (2015, unpubil. data).

**Table 2:** Morphological characteristics and AFLP genotypes of *Sporothrix globosa* isolates in China

**Group by AFLP**	**Number of isolates**	**Mean colony diameter (mm)±SD**			**Growth rate (mm/week)±SD**		
		**30 °C**	**35 °C**	**37 °C**	**30 °C**	**35 °C**	**37 °C**
I	52	29.89±5.02^a^	6.48±2.16^a^	3.12±1.17^a^	9.96±1.67^a^	2.16±0.27^a^	1.04±0.39^a^
II	93	31.86±5.02^a^	8.58±2.20^b^	2.54±0.63^b^	10.62±1.67^a^	2.86±0.73^b^	0.85±0.21^b^
III	2	32.00±2.83^a,b^	9.50±0.71^a,b^	4.95±0.07^c^	10.67±0.94^a,b^	3.17±0.24^a,b^	1.65±0.02^c^
IV	49	34.53±3.25^b^	9.21±2.34^b^	2.56±0.72^a,b^	11.51±1.08^b^	3.07±0.78^b^	0.85±0.24^a,b^
V	12	32.73±6.04^a,b^	9.67±3.22^b^	3.20±1.15^a,b^	10.91±2.01^a,b^	3.22±1.07^b^	1.07±0.38^a,b^
VI	2	34.25±1.06^a,b^	8.25±2.47^a,b^	2.40±0.28^a,b,c^	11.42±0.35^a,b^	2.75±0.82^a,b^	0.80±0.09^a,b,c^
VII	3	33.83±5.80^a,b^	10.3±0.58^a,b^	3.23±0.84^a,b,c^	11.28±1.93^a,b^	3.44±0.19^a,b^	1.08±0.28^a,b,c^
VIII	3	25.83±3.75^a,b^	6.17±0.29^a,b^	2.97±0.35^a,b^	8.61±1.25^a,b^	2.06±0.10^a,b^	0.99±0.12^a,b^
total	216	31.9±4.97	8.26±2.49	2.76±0.90	10.67±1.66	2.75±0.83	0.92±0.30

*α*=0.05; a, b, c=Groups; Nine isolates were excluded since they were not clustered into the AFLP groups.

**Table 3:** Distribution of *Sporothrix globosa* AFLP genotypes among different geographic origins in China

	**Group I**		**Group II**				**Group III**	**Group IV**				**Group V**	**Group VI**	**Group VII**	**Group VIII**	**Total**
	**Ia**	**Ib**	**IIa**	**IIb**	**IId**	**IIe**		**IVa**	**IVb**	**IVc**	**IVd**					
Jilin Province																
Changchun			27	2	2	4		17	4	4						60
Songyuan	17	1	5		2		2	10							3	40
Siping			5	18				4			1	1		2		31
Jilin	11	7											1			19
Baicheng	12															12
Liaoyuan	2		2	4							1		1	1		11
Tonghua			5					3								8
Baishan	2											1				3
Yanbian			1					2				1				4
Heilongjiang			1									1				2
Neimenggu			1					2								3
Beijing												3				3
Jiangsu			2					1				2				5
Sichuan			3									1				4
Chongqing			7									1				8
Guangdong			2									1				3
Total	44	8	61	24	4	4	2	39	4	4	2	12	2	3	3	216
	52		93					49								

**Table 4:** Relationship between AFLP genotypes and different clinical forms

	**Group I**	**Group II**	**Group III**	**Group IV**	**Group V**	**Group VI**	**Group VII**	**Group VIII**	**Total**
*Clinical forms*^a^									
F	26	46	2	31	4	1		2	112
L	22	31		16		1	3	1	74
D	4	1		1					6
E		1							1

a F—fixed cutaneous; L—lymphocutaneous; D—disseminated cutaneous; E—extracutaneous.

**Table 5:** Susceptibility testing results in μg/mL of *Sporothrix globosa* isolates

**Group by AFLP**	**MIC**	**FCZ**	**ICZ**	**VCZ**	**TRB**	**AMB**	**POS**	**CAS**	**ALB**
I (*n*=10)	Range	64–>64	2–>16	8–>16	0.03–8	>16	0.5–>16	0.5–>16	4–16
	GM	>64	12.12	>16	0.06	>16	3.03	6.06	7.46
II (*n*=10)	Range	>64	2–>16	8–>16	0.03–0.06	>16	1–>16	8–>16	4–16
	GM	>64	9.19	>16	0.03	>16	2.14	>16	8
III (*n*=2)	Range	>64	8–>16	16–>16	0.03	>16	1–4	0.25–16	4–16
	GM	—	—	—	—	—	—	—	—
IV (*n*=9)	Range	>64	2–>16	>16	0.03–0.5	>16	1–16	8–>16	4–16
	GM	>64	12.70	>16	0.07	>16	2	>16	8.64
V (*n*=4)	Range	>64	2–>16	16–>16	0.03–0.06	>16	1–>16	1–16	8–16
	GM	>64	9.51	>16	0.04	>16	6.72	4.76	11.31
VI (*n*=2)	Range	>64	>16	>16	0.03–0.25	>16	>16	4–>16	8–16
	GM	—	—	—	—	—	—	—	—
VII (*n*=3)	Range	>64	16–>16	16–>16	0.03–0.06	>16	1–>16	1–16	8–16
	GM	>64	>16	>16	0.04	>16	4	2.52	10.08
VIII (*n*=3)	Range	>64	1–16	16–>16	0.03	>16	1–8	1–>16	4–8
	GM	>64	6.35	>16	0.03	>16	2	8	5.04
Total (*n*=43)	Range	>64	1–>16	8–>16	0.03–8	>16	0.5–>16	0.25–>16	4–16
	GM	>64	11.78	>16	0.05	>16	2.99	9.55	8.26

Abbreviations: amplified fragment length polymorphism, AFLP; albaconazole, ALB; amphotericin B, AMB; caspofungin, CAS; fluconazole, FCZ; genometric mean, GM; itraconazole, ICZ; minimum inhibitory concentrations, MIC; posaconazole, POS; terbinafine, TRB.
